# Pyruvate Upregulates Hepatic FGF21 Expression by Activating PDE and Inhibiting cAMP–Epac–CREB Signaling Pathway

**DOI:** 10.3390/ijms23105490

**Published:** 2022-05-14

**Authors:** Yan-Yan Zhao, Li-Jun Zhang, Xiang-Yan Liang, Xiao-Chun Zhang, Jin-Rui Chang, Man Shi, Huan Liu, Ying Zhou, Zhuo Sun, Yu-Feng Zhao

**Affiliations:** Institute of Basic Medical Sciences, Xi’an Medical University, Xi’an 710021, China; zhaoyanyan@xiyi.edu.cn (Y.-Y.Z.); zhanglijun@xiyi.edu.cn (L.-J.Z.); liangxiangyan@xiyi.edu.cn (X.-Y.L.); zhangxiaochun@xiyi.edu.cn (X.-C.Z.); changjinrui@xiyi.edu.cn (J.-R.C.); manshi@xiyi.edu.cn (M.S.); liuhuan@xiyi.edu.cn (H.L.); zhouying209@xiyi.edu.cn (Y.Z.); sunzhuo@xiyi.edu.cn (Z.S.)

**Keywords:** pyruvate, FGF21, hepatocytes, PDE, cAMP, CREB

## Abstract

Fibroblast growth factor 21 (FGF21) functions as a polypeptide hormone to regulate glucose and lipid metabolism, and its expression is regulated by cellular metabolic stress. Pyruvate is an important intermediate metabolite that acts as a key hub for cellular fuel metabolism. However, the effect of pyruvate on hepatic FGF21 expression and secretion remains unknown. Herein, we examined the gene expression and protein levels of FGF21 in human hepatoma HepG2 cells and mouse AML12 hepatocytes in vitro, as well as in mice in vivo. In HepG2 and AML12 cells, pyruvate at concentrations above 0.1 mM significantly increased FGF21 expression and secretion. The increase in cellular cAMP levels by adenylyl cyclase activation, phosphodiesterase (PDE) inhibition and 8-Bromo-cAMP administration significantly restrained pyruvate-stimulated FGF21 expression. Pyruvate significantly increased PDE activities, reduced cAMP levels and decreased CREB phosphorylation. The inhibition of exchange protein directed activated by cAMP (Epac) and cAMP response element binding protein (CREB) upregulated FGF21 expression, upon which pyruvate no longer increased FGF21 expression. The increase in plasma pyruvate levels in mice induced by the intraperitoneal injection of pyruvate significantly increased FGF21 gene expression and PDE activity with a reduction in cAMP levels and CREB phosphorylation in the mouse liver compared with the control. In conclusion, pyruvate activates PDEs to reduce cAMP and then inhibits the cAMP–Epac–CREB signaling pathway to upregulate FGF21 expression in hepatocytes.

## 1. Introduction

The hepatocyte plays a central role in fuel and energy metabolism, and it secretes hormonal signals to modulate the metabolism in response to different nutritional statuses [1,2,3]. Fibroblast growth factor 21 (FGF21) is a hepatocyte-secreted hormone and has broad metabolism-regulating actions [4,5]. FGF21 stimulates lipolysis and fatty acid oxidation, enhances ketone body production and utilization, induces the weight loss of obese rodents and primates and protects many tissues and organs against stress damage [6,7,8,9]. FGF21 is a promising drug candidate for the treatment of metabolic diseases, such as type 2 diabetes, obesity and nonalcoholic steatohepatitis [10].

Pyruvate is a pivotal glucose metabolite that resides at the intersection of glycolysis and oxidative phosphorylation [11]. It enters mitochondria for aerobic oxidation in the presence of oxygen to generate energy, while it is reduced into lactate in the cytoplasm in the absence of oxygen. On the other hand, pyruvate can be interchangeably converted to oxaloacetate and alanine, which are involved in the process of gluconeogenesis and protein synthesis. Meanwhile, pyruvate is a scavenger of reactive oxygen species (ROS) and protects the cells against oxidative damage. Thus, the exogenous supplementation with pyruvate has many biological effects. For example, it resulted in weight loss in obese (fa/fa) Zucker rats [12], and induced weight and fat loss in hyperlipidemia patients [13]. Combined supplementation with pyruvate and creatine increases lean body mass and enhances training adaptation in American football players [14]. In addition, pyruvate improves ischemia-reperfusion injury in the intestine, heart and brain [15,16,17,18,19,20], which may be attributed to its antioxidative function since pyruvate is able to scavenge H_2_O_2_ [21,22,23,24]. ROS can lead to mitochondrial damage and influence energy generation, and the cleavage of ROS by pyruvate may benefit cell fuel and energy metabolism. Since pyruvate is pivotal in regulating cell metabolism, it may regulate hepatic FGF21 expression, the metabolism-related hormone. However, the effect of pyruvate on hepatic FGF21 expression and secretion remains unknown.

Hepatic FGF21 expression is finely regulated by hormones, nutrients and the cell stress-inducing factors, which include glucagon, thyroid hormone, lipoic acid, retinoic acid and curcumin [5,25]. Additionally, multiple intracellular signaling molecules, such as peroxisome proliferator activator receptor-α (PPAR-α), AMP-activated kinase (AMPK) and activating transcriptional factors (ATFs), have been demonstrated to regulate hepatic FGF21 expression [26,27,28,29]. Meanwhile, other signaling molecules, such as cAMP and CREB, are indicated to regulate FGF21 expression. For instance, glucagon activates hepatic cAMP signaling and inhibits FGF21 gene expression [30]. CREB-regulated transcription co-activator 2 (CRTC2) liver-specific knockout mice display significant increases in plasma and hepatic FGF21 levels, which suggests that CREB/CRTC2 negatively regulates FGF21 expression [31]. Adenylyl cyclase (AC) catalyzes the production of cAMP from ATP while phosphodiesterases (PDEs) hydrolyzes cAMP to AMP, and they coordinately control intracellular cAMP levels and may be involved in the regulation of FGF21 expression [32,33]. To understand the mechanism by which pyruvate regulates metabolism, we observed the effect of pyruvate on hepatic FGF21 expression and secretion in hepatocytes and explored its corresponding signaling pathway in this study.

## 2. Results

### 2.1. Pyruvate Upregulated FGF21 Expression and Secretion in HepG2 Cells

**Figure 1:**
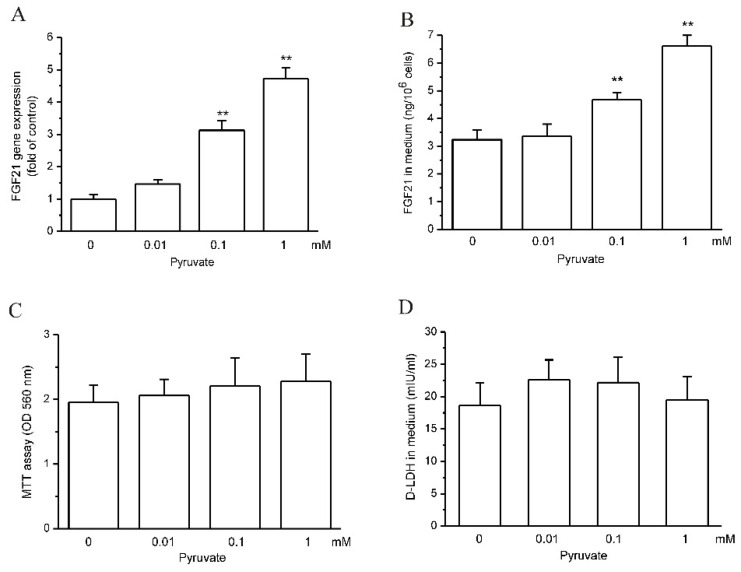
Pyruvate upregulated FGF21 expression and secretion in HepG2 cells. (**A**): pyruvate dose-dependently stimulated FGF21 gene expression after 12 h treatment in HepG2 cells (** *p* < 0.01 vs. control, *n* = 3). (**B**): FGF21 protein levels in cell medium significantly increased after pyruvate treatment (** *p* < 0.01 vs. control, *n* = 10). (**C**): The cell viability was not influenced by pyruvate treatment, as shown by MTT assay (*n* = 10). (**D**): D-LDH levels in cell medium were not changed by pyruvate treatment (*n* = 10).

As shown by RT-qPCR, pyruvate upregulated FGF21 gene expression after 12 h treatment from 0.1 mM and reached stronger effects at 1 mM (*p* < 0.01; *n* = 3; Figure 1A). FGF21 protein levels in cell medium also significantly increased after pyruvate treatment, which is in accordance with FGF21 gene expression (*p* < 0.01; *n* = 10; Figure 1B). The cell viability was not influenced by pyruvate in 12 h treatment below 1 mM, as shown by MTT assay (Figure 1C). The LDH levels in medium also did not show differences after pyruvate treatment compared with the control, which further confirmed that pyruvate was not toxic to HepG2 cells below 1 mM, and FGF21 in cell medium was derived from increased cell secretion (Figure 1D).

### 2.2. Pyruvate Activated PDEs and Reduced cAMP to Upregulate FGF21 Expression in HepG2 Cells

**Figure 2:**
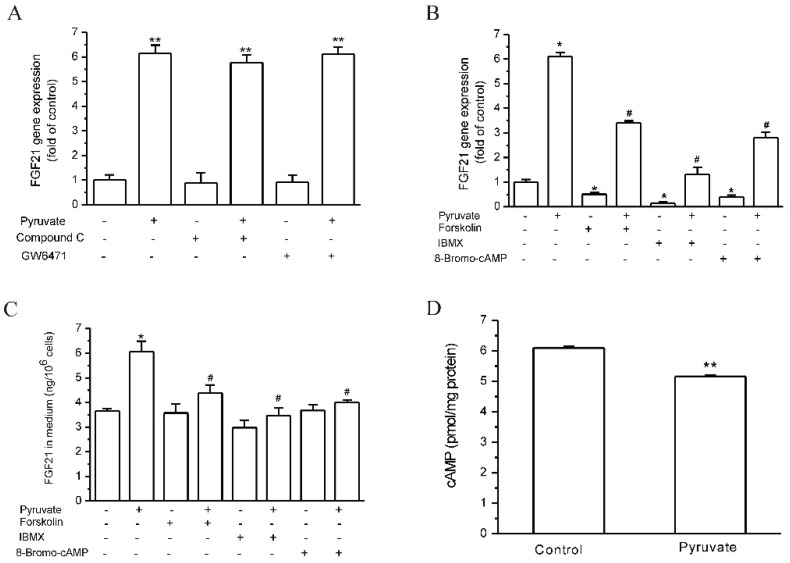
cAMP reduction caused pyruvate-stimulated FGF21 expression in HepG2 cells. (**A**): The activation of PPAR-α and AMPK was not involved in pyruvate-stimulated FGF21 expression (** *p* < 0.01 vs. control, *n* = 3). (**B**): AC activator forskolin, PDE inhibitor IBMX and 8-Bromo-cAMP administration significantly inhibited FGF21 expression and suppressed pyruvate-stimulated FGF21 expression (* *p* < 0.05 vs. control, # *p* < 0.05 vs. pyruvate group, *n* = 3). (**C**): Forskolin, IBMX and 8-Bromo-cAMP inhibited pyruvate-stimulated increase in FGF21 protein levels in cell medium (* *p* < 0.05 vs. control, # *p* < 0.05 vs. pyruvate group, *n* = 10). (**D**): Pyruvate decreased intracellular cAMP levels in HepG2 cells (** *p* < 0.01 vs. control, *n* = 12).

Pyruvate is involved in cell metabolism, and PPAR-α and AMPK are two signaling molecules that are closely related to hepatocyte metabolism and FGF21 expression [28,29]. We first observed the effects of PPAR-α and AMPK on pyruvate-regulated FGF21 expression in HepG2 cells, and it was found that both PPAR-α inhibition by GW6471 and AMPK inhibition by Compound C had no significant effect on pyruvate-regulated FGF21 expression (Figure 2A). In the following studies of the signaling pathways for pyruvate-regulated FGF21 expression, we found that both AC activator forskolin and PDE inhibitor IBMX inhibited FGF21 expression (*p* < 0.05; *n* = 3; Figure 2B), and they suppressed pyruvate-stimulated FGF21 expression (*p* < 0.05; *n* = 3; Figure 2B). In addition, 8-Bromo-cAMP, which is the cell-permeable cAMP analog and elevates cytosolic cAMP levels, showed the same effects as forskolin and IBMX on FGF21 expression (*p* < 0.05; *n* = 3; Figure 2B). Meanwhile, forskolin, IBMX and 8-Bromo-cAMP significantly reduced the pyruvate-stimulated increase in FGF21 protein levels in culture medium, although they did not show a significant inhibition of FGF21 protein levels by themselves (*p* < 0.05; *n* = 10; Figure 2C). These results indicate that pyruvate may decrease the intracellular cAMP levels to stimulate FGF21 expression and secretion. The intracellular cAMP levels in HepG2 cells were then measured, and it was found that pyruvate significantly decreased intracellular cAMP levels in HepG2 cells (*p* < 0.01; *n* = 12; Figure 2D). Therefore, the reduction in intracellular cAMP levels is suggested to be the reason for pyruvate-stimulated FGF21 expression and secretion in HepG2 cells.

**Figure 3:**
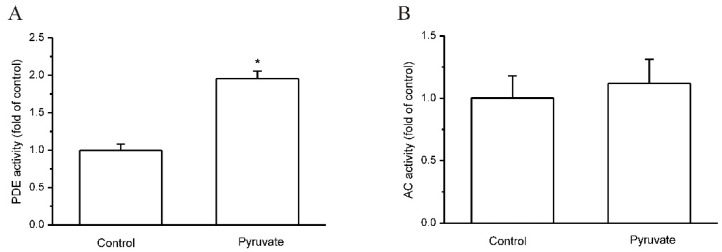
Pyruvate activated PDE in HepG2 cells. (**A**): Pyruvate significantly increased phosphodiesterase (PDE) activities in HepG2 cells (* *p* < 0.05 vs. control, *n* = 5). (**B**): Pyruvate had no effect on adenylyl cyclase (AC) activities in HepG2 cells (*n* = 5).

The intracellular cAMP level is determined by the balance between AC-catalyzed cAMP production and PDE-catalyzed cAMP hydrolysis. The activities of AC and PDE in HepG2 cells were then measured, and it showed that pyruvate significantly increased PDE activities in HepG2 cells (*p* < 0.05; *n* = 5; Figure 3A). In contrast, AC activities were not influenced by pyruvate in HepG2 cells (Figure 3B).

### 2.3. Epac and CREB Were Involved in Pyruvate-Stimulated FGF21 Expression in HepG2 Cells

**Figure 4:**
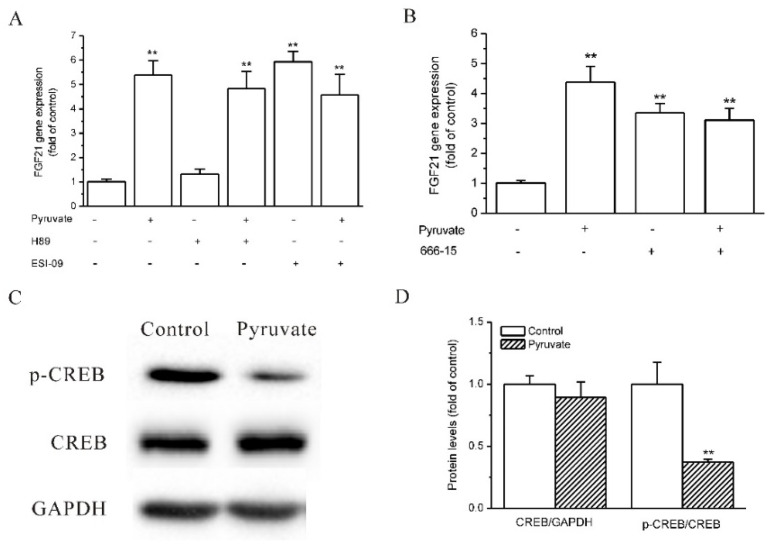
Epac and CREB were involved in pyruvate-stimulated FGF21 expression in HepG2 cells. (**A**): Epac inhibitor ESI-09 but not PKA inhibitor H89 upregulated FGF21 expression and eliminated the stimulatory effect of pyruvate on FGF21 expression (** *p* < 0.01 vs. control, *n* = 3) (**B**): CREB inhibitor 666-15 upregulated FGF21 expression and eliminated the stimulatory effect of pyruvate on FGF21 expression (** *p* < 0.01 vs. control, *n* = 3). (**C**,**D**): Pyruvate reduced CREB phosphorylation without influencing the total CREB protein levels (** *p* < 0.01 vs. control, *n* = 3).

The downstream molecules of the cAMP signaling pathway that mediate pyruvate-regulated FGF21 expression were then investigated. Protein kinase A (PKA) and exchange protein directed activated by cAMP (Epac) are two critical molecules that are directly activated by cAMP, and CREB is the transcriptional factor that regulates gene expression in response to cAMP [34,35]. The role of PKA, Epac and CREB in pyruvate-stimulated FGF21 expression was observed. The Epac inhibitor ESI-09 upregulated FGF21 expression as pyruvate did, and it eliminated the stimulatory effects of pyruvate on FGF21 expression in HepG2 cells (*p* < 0.01; *n* = 3; Figure 4A). PKA inhibitor H89 did not influence pyruvate-regulated FGF21 expression (Figure 4A). CREB inhibitor, 666-15, stimulated FGF21 expression and eliminated the stimulatory effects of pyruvate on FGF21 expression in HepG2 cells (*p* < 0.01; *n* = 3; Figure 4B). This indicates that Epac and CREB inhibit FGF21 expression and pyruvate decreases Epac and CREB signaling to increase FGF21 expression in HepG2 cells. CREB phosphorylation was then observed, and Western blot showed that although the total CREB protein levels were not different between pyruvate treatment and the control, pyruvate significantly decreased CREB phosphorylation in HepG2 cells (*p* < 0.01; *n* = 3; Figure 4C,D).

### 2.4. Pyruvate Stimulated FGF21 Expression in Mouse AML-12 Hepatocytes and Mouse Liver In Vivo

**Figure 5:**
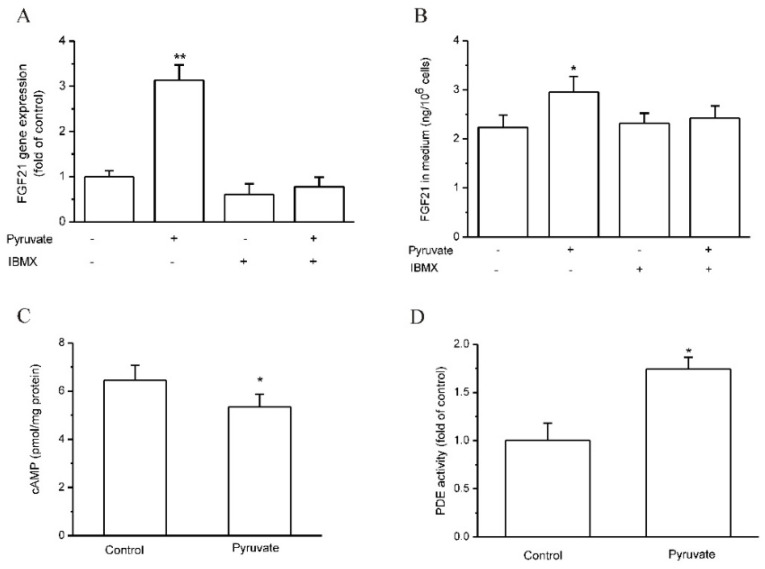
Pyruvate upregulated FGF21 expression and secretion in mouse hepatic AML-12 cells. (**A**,**B**): Pyruvate stimulated FGF21 expression and secretion in AML-12 cells (* *p* < 0.05 and ** *p* < 0.01 vs. control, *n* = 6). (**C**): Pyruvate decreased intracellular cAMP levels in AML12 cells (* *p* < 0.05, *n* = 5). (**D**): Pyruvate increased PDE activities in AML-12 cells (* *p* < 0.05, *n* = 5).

**Figure 6:**
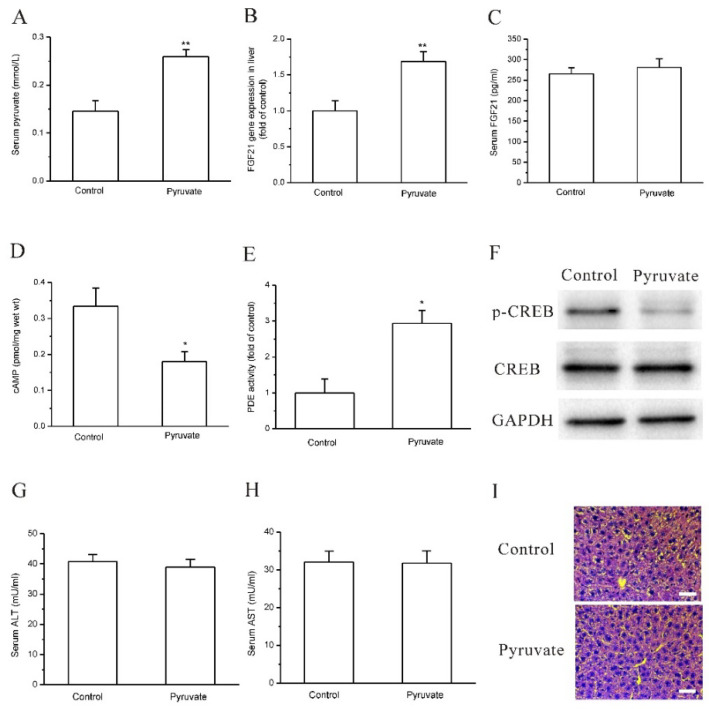
Pyruvate upregulated FGF21 expression in mice in vivo. (**A**): Intraperitoneal injection of pyruvate significantly increased serum pyruvate levels in C57BL/6J mice compared with the control (** *p* < 0.01, *n* = 10). (**B**): The pyruvate-treated mice had significantly higher FGF21 gene expression in liver than the control (** *p* < 0.01, *n* = 10). (**C**): Serum FGF21 levels were not changed by pyruvate treatment in mice compared with the control (*n* = 10). (**D**): cAMP levels in mouse liver were significantly decreased by pyruvate treatment in mice (* *p* < 0.05, *n* = 10). (**E**): PDE activity in mouse liver was significantly activated by pyruvate injection in mice (* *p* < 0.05, *n* = 10). (**F**): CREB phosphorylation was inhibited in liver after pyruvate injection compared with the control. (**G**,**H**): ALT and AST activities in mouse serum were not significantly different between pyruvate-treated group and the control group. (**I**): H&E staining showed that the liver tissues had normal morphology in mice of pyruvate-treated group without obvious difference to the control (bar is 20 μm).

We next attempted to demonstrate whether pyruvate upregulates FGF21 expression in vivo in mice. Before that, the effects of pyruvate on mouse hepatocytes were observed. In mouse AML-12 hepatocytes, pyruvate (1mM) significantly stimulated FGF21 expression and secretion, which was significantly suppressed by IBMX (*p* < 0.05; *n* = 6; Figure 5A,B). Pyruvate also significantly increased PDE activities and lowered cAMP levels in AML-12 hepatocytes (*p* < 0.05; *n* = 5; Figure 5C,D). The results indicated that pyruvate had an effect on FGF21 expression in mouse hepatocytes. Then, pyruvate was administrated via intraperitoneal injection in C57BL/6J mice. The serum levels of pyruvate were significantly higher in pyruvate-treated mice than the control mice with PBS injection (*p* < 0.015; *n* = 10; Figure 6A). The pyruvate-treated mice had significantly higher FGF21 gene expression in the liver than the control (*p* < 0.01; *n* = 10; Figure 6B). Serum FGF21 protein levels were not significantly different between pyruvate-treated mice and the control (Figure 6C). PDE activity in the mouse liver was significantly activated, and cAMP levels significantly decreased after pyruvate treatment compared with the control (*p* < 0.05; *n* = 10; Figure 6D,E). CREB phosphorylation was also significantly inhibited in the mouse liver after pyruvate treatment compared with the control (*p* < 0.05; *n* = 10; Figure 6F). To determine whether the toxic effect of pyruvate exists, the glutamic-pyruvic transaminase (ALT) and glutamic-oxaloacetic transaminase (AST) activities in mouse serum were measured and they were not significantly different between the two groups (Figure 6G,H). Hematoxylin-eosin (H&E) staining showed that the liver tissues had normal morphology in the mice of the pyruvate-treated group without obvious difference to the control (Figure 6I).

## 3. Discussion

This study demonstrates that pyruvate upregulates FGF21 expression and secretion in hepatocytes in vitro and in vivo. Pyruvate is a hub of cellular fuel metabolism and used as a supplement in cell culture [36,37,38], and the most commonly used concentration is 1 mM in culture medium. This indicates that a concentration of pyruvate below 1 mmol/L is safe for cells. Pyruvate upregulated FGF21 expression in HepG2 cells and AML-12 cells at a concentration of below 1 mM in vitro, at which it did not display toxicity to these two kinds of hepatocytes. Therefore, the effect of pyruvate on FGF21 expression is due to its normal regulation of cellular metabolism or intracellular signaling pathways.

Subsequently, we explored the mechanism of pyruvate-stimulated FGF21 expression. Since pyruvate is important in fuel metabolism, we first observed the role of AMPK and PPAR-α, two important signaling molecules that are activated in response to changes in metabolism and are well known regulators of FGF21 expression. However, AMPK and PPAR-α inhibition did not influence pyruvate-stimulated FGF21 expression, which indicates that AMPK and PPAR-α do not mediate the effect of pyruvate on FGF21 expression. On the other hand, it was unexpectedly found that the PDE inhibition of IBMX, cAMP analogue and AC activation by forskolin, all of which increase intracellular cAMP levels, inhibited FGF21 gene expression and suppressed the effects of pyruvate on FGF21 gene expression. These results indicate that pyruvate induces a decrease in cAMP levels to upregulate FGF21 expression in hepatocytes.

Intracellular cAMP levels decreased in hepatocytes with pyruvate treatment, as shown by the cAMP measurement. The intracellular cAMP level is controlled by the activity of AC and PDEs, which catalyze cAMP production and cAMP hydrolysis, respectively [32,33,39]. The enzyme activity assay showed that pyruvate increased PDE activity and had no effect on AC activity. It is concluded that pyruvate decreases intracellular cAMP levels by activating PDEs in hepatocytes. The complete failure of pyruvate to upregulate FGF21 expression under PDE inhibition by IBMX supports this conclusion. To the best of our knowledge, this is the first report showing that pyruvate activates PDE and decreases intracellular cAMP levels. The PDE superfamily includes 11 enzymes (PDE1-11) that hydrolyze cAMP and cGMP, and at least six PDEs (PDE1, 2, 3, 4, 7 and 8) hydrolyze cAMP and most of them are expressed in the liver [33,40,41,42,43]. The present study did not identify the concrete member(s) of PDEs that are activated by pyruvate, which remains to be fully studied in the future. Another inadequacy of this study is that the activating mechanism of PDEs by pyruvate has not been demonstrated, although direct interactions of pyruvate with PDEs were suggested as pyruvate activated PDEs in the homogenates of the cells and liver tissue in this study.

The downstream molecules in the cAMP signaling pathway that are involved in pyruvate-stimulated FGF21 expression were analyzed, which indicated that Epac and CREB mediate the effect of pyruvate on FGF21 expression. PKA and Epac are two key molecules that are directly activated by cAMP, which in turn elicits the signaling cascade and then activates CREB by enhancing its phosphorylation [34,35]. Many cellular responses to cAMP are mediated in parallel by PKA and Epac [44,45,46]. Meanwhile, Epac and PKA are able to mediate the effects of cAMP signaling independently in many kinds of cells [47,48,49]. In this study, we found that Epac inhibition but not PKA inhibition mimicked and eliminated the effects of pyruvate on FGF21 expression. Therefore, the inhibition of the Epac signaling pathway is responsible for pyruvate-regulated FGF21 expression. CREB is the transcriptional factor that binds to the cAMP-response element and regulates gene expression [50,51]. In this study, CREB inhibition upregulated FGF21 expression and eliminated the stimulatory effects of pyruvate on FGF21 expression. Therefore, CREB is the signaling molecule that is inhibited following a reduction in cAMP, which is further confirmed by the significant decrease in the phosphorylation of CREB in HepG2 cells and in mouse liver under pyruvate treatment. It has been well known that Epac signaling activation stimulates CREB phosphorylation to regulate gene expression [52,53,54]. Taken together, the present study demonstrates that pyruvate activates PDEs to reduce intracellular cAMP and then inhibits the cAMP–Epac–CREB signaling pathway to upregulate FGF21 expression in hepatocytes, which extends the understanding of the regulatory mechanism of FGF21 expression.

The pyruvate-induced inhibition of the cAMP signaling pathway may be functional in the regulation of the fuel metabolism. It was reported that pyruvate content was elevated from 9.88 nmol/g wet wt to 18.76 nmol/g wet wt in rat adipose tissue after 20 min incubation with 10 mM glucose [55]. Glucose administration to 24 h fasted rats increased pyruvate content from 25 nmol/g wet wt to 104 nmol/g wet wt in the liver [56]. It is well known that cAMP signaling that is activated by glucagon and epinephrine in hepatocytes stimulates glycogen hydrolysis to maintain blood glucose levels during fasting and exercise [57,58]. Therefore, to prevent the excessive elevation of blood glucose levels, pyruvate increases in the liver after glucose uptake and then inhibits cAMP signaling to reduce hepatic glucose production.

It has been shown that the intermediate metabolites of carbohydrates, lipids and amino acids are able to regulate the intracellular signaling transduction and gene expression. For instance, lactate inhibits histone deacetylases activity [59]; phosphoenolpyruvate inhibits the sarcoplasmic reticulum Ca^2+^-ATPase (SERCA)- Ca^2+^-nuclear factor of the activated T-cell (NFAT) signaling pathway [60,61]; Acyl-CoA activates PPAR-β [62]. Little is known about the functional regulation of PDEs by intermediate metabolites. The present study demonstrates that pyruvate activates PDEs in hepatocytes, which enriches the relationships between intracellular signaling pathways and the intermediate metabolites.

The effects of pyruvate on FGF21 expression were further confirmed in vivo in mice. After mouse blood pyruvate levels were elevated via the intraperitoneal injection of pyruvate, mouse liver cAMP levels decreased and FGF21 gene expression increased compared with the control. Levels of pyruvate can stay stable for a long time in vitro, but it is quickly metabolized in vivo and is unable to sustain a high level for a long time. Therefore, the increasing level of FGF21 mRNA in the liver was less than that observed in vitro. The blood FGF21 protein level did not significantly increase in pyruvate-treated mice compared with the control, which further suggested that exogenous pyruvate administration had less of an effect on hepatic FGF21 expression and secretion in vivo than in vitro. Although pyruvate provides energy for the brain and enhances neurological recovery from cardiopulmonary arrest-induced damage, a rapid injection of high-dose pyruvate leads to respiratory arrest and even death in mice [63,64]. A recent study shows that a single injection of pyruvate induces torpor in obese mice [65]. In accordance with these previous reports, the present study also found that pyruvate injection induced torpor and poor health in mice. Therefore, the systemic elevation of pyruvate via the intravenous or intraperitoneal infusion of pyruvate is not a feasible approach to regulating hepatic FGF21 expression. The elevation of blood pyruvate did not result in liver damage because the activities of ALT and AST in blood were not significantly increased and the liver tissues had normal morphology after the intraperitoneal infusion of pyruvate in mice. It is suggested that pyruvate leads to neuronal dysfunction and induced torpor in mice without damage to liver. Thus, the approaches that specifically increase pyruvate levels in hepatocytes to stimulate FGF21 expression may warrant further exploration in future study.

**Figure 7:**
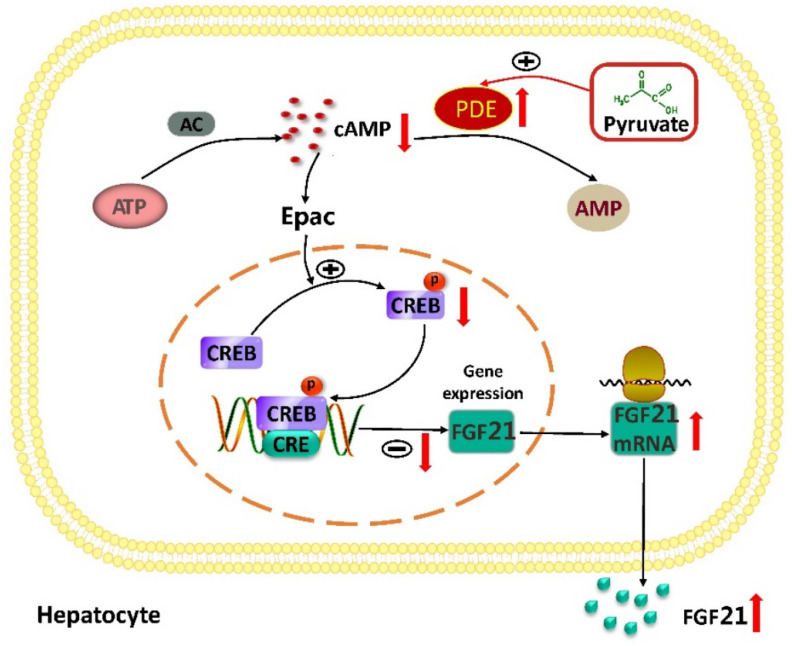
The diagram of pyruvate-stimulated FGF21 expression in hepatocytes. cAMP–Epac–CREB signaling inhibits FGF21 expression in human and mouse hepatocytes. Pyruvate activates PDEs to reduce cAMP levels and then inhibits cAMP–Epac–CREB signaling to upregulate FGF21 expression in hepatocytes.

As summarized in Figure 7, this study demonstrates that pyruvate activates PDEs and inhibits cAMP–Epac–CREB signaling to stimulate FGF21 expression in human and mouse hepatocytes. This finding provides a new regulatory mechanism of hepatic FGF21 expression and links pyruvate to the cAMP signaling pathway in hepatocytes. Given that FGF21 improves metabolic disorders, this study indicates that the strategies to increase intracellular pyruvate levels in hepatocytes may benefit form metabolic disorder therapy.

## 4. Materials and Methods

### 4.1. Materials

Pyruvate, Compound C, GW6471, forskolin, IBMX, 8-Bromo-cAMP, H89, ESI-09, 666-15 and MTT were purchased from Merck KGaA (Darmstadt, Germany). Dulbecco’s modified eagle medium (DMEM), fetal bovine serum (FBS) and trypsin for cell culture were obtained from Thermo Fisher (Waltham, MA, USA). The kits for RNA extractive, reverse transcription (RT) and quantitative PCR (qPCR) were obtained from Takara Bio Inc (Dalian, China). Protein extraction kits and BCA assay kits were purchased from Bio-Rad Laboratories (Hercules, USA). Human and mouse FGF21 ELISA kits, cAMP assay kits, the ALT activity assay kit, the AST activity assay kit and the LDH assay kit were obtained from Elabscience (Wuhan, China). Pyruvate assay kits were obtained from Abcam (Boston, MA, USA). Anti-cAMP response element-binding protein (CREB) and anti-phospho-CREB (Ser133) antibodies were obtained from Cell Signaling Technology (Beverly, MA, USA). H&E staining kits were obtained from Beyotime Biotechnology (Beijing, China).

### 4.2. Cell Culture

Human hepatoma HepG2 cell and mouse AML12 hepatocyte were obtained from American Type Culture Collection (ATCC, Washington, DC, USA) and cultured in DMEM containing 5.6 mM glucose, 1mM sodium pyruvate and 10% FBS. The media were refreshed using 10% DMEM without sodium pyruvate the day before drug treatment.

### 4.3. RT-qPCR

Total RNA extraction and RT were performed using the kits, and FGF21 gene expression was measured using qPCR with SYBR premix EX Taq. Relative gene expression was calculated using the comparative cycle threshold (Ct) value normalized to GAPDH, and the 2^−ΔΔCt^ method was used to reflect the levels of gene expression. The primers were as follows: human FGF21 forward 5′-GGG AGT CAA GAC ATC CAG GT-3′ and reverse 5′-GGC TTC GGA CTG GTA AAC AT-3′; human GAPDH forward 5′- GAC AAC TTT GGT ATC GTG G-3′ and reverse 5′-AGG CAG GGA TGA TGT TCT-3′; mouse FGF21 forward 5′-TCC AAA TCC TGG GTG TCA AA-3′ and reverse 5′-CAG CAG CAG TTC TCT GAA GC-3′; mouse GAPDH forward 5′-GAG AAC TTT GGC ATT GTG G-3′ and reverse 5′-ATG CAG GGA TGA TGT TCT G-3′.

### 4.4. ELISA

FGF21 levels in culture medium and mouse serum were measured using ELISA kits. Briefly, the samples and the standards were added to 96-well plates in duplicate for incubation at 37 °C for 1.5 h. Then, the biotinylated detection antibodies were added for 1 h incubation at 37 °C. The plates were washed with PBS 3 times, and then avidin-HRP conjugates were added for 30 min incubation at 37 °C. The plates were washed 5 times, and the substrate reagent was added to each well for color development at 37 °C for 10 min. After the stop solution was added, the optical density (OD) values of each well were measured at 450 nm. The standard curve was established, and FGF21 levels were calculated for the samples. The LDH levels in the culture medium were measured using LDH ELISA kits in the same procedure as the FGF21 assay.

### 4.5. Cell Viability Assay

When HepG2 cells were changed to serum-free medium and treated by pyruvate for 12 h, they reached up to 70% confluence in 96-well plates, and then MTT was added into the medium (0.5mg/mL in final) for 4 h incubation. The medium was discarded, and 100 μL acidic isopropanole was added to each well to dissolve MTT crystals. The OD values at 560 nm were measured.

### 4.6. Western Blot

Total proteins were extracted using ReadyPrepTM Protein extraction kits and quantified using BCA assay. The protein samples were electrophorized and transferred to a nitrocellulose membrane. Whilst washing with TBS between the steps, the membranes were incubated in sequences by blocking buffer for 2 h at room temperature (RT), rabbit-anti-CREB antibody (1:1000) or rabbit-anti-phospho-CREB antibody (1:1000) at 4 °C overnight, HRP-conjugated goat-anti-rabbit IgG antibodies for 1 h at RT and chemiluminescent substrates at RT. Then, the luminescence was imaged using the ChemiDoc MP gel imaging system.

### 4.7. cAMP Measurement

A competitive ELISA method was used to measure cAMP levels in accordance with the kit instructions. Briefly, samples (50 μL per well) and biotinylated detection antibody (50 μL per well) were added into cAMP pre-coated wells for 45 min incubation at 37 °C. The plate was washed, and then HRP-conjugate antibodies (100 μL per well) were added for 30 min incubation at 37 °C. After the plate was washed, substrate solution (90 μL per well) was added for 15 min at 37 °C. Finally, stop solution (50 μL per well) was added, and the OD values at 450 nm were measured to calculate cAMP levels.

### 4.8. Adenylyl Cyclase (AC) and Phosphodiesterases (PDEs) Activity Assay

The homogenates of HepG2 cells, AML12 cells and mouse liver were prepared using a physical lysing method with FastPrep-24 homogenizer (MP Biomedicals, Santa Ana, CA, USA). The lysing buffer is composed of (mmol/L) NaCl 10, KCl 140, MgSO_4_ 10, Na_2_HPO_4_ 1.2, KH_2_PO_4_ 0.2, glucose 5 and HEPES 10 (pH 7.35). The homogenates were prepared at 10^8^ cells/mL lysing buffer for HepG2 and AML12 cells and at 100mg/mL lysing buffer for mouse liver. For AC activity assay, ATP (1 mg/mL) and IBMX (200 μmol/L) were added, followed by pyruvate (1 mmol/L) or placebo (lysing buffer) and left to react for 1 h at 37 °C. Then, the enzymes were inactivated by heating at 95 °C for 3 min, and the cAMP levels were measured to evaluate AC activity. For PDE activity assay, pyruvate (1 mmol/L) or placebo (lysing buffer) was added into the homogenates, and then 50 ng/mL cAMP was added and left to react for 1 h at 37 °C. The enzymes were inactivated by heating at 95 °C for 3 min, and cAMP levels were measured to evaluate PDE activity.

### 4.9. Animal Experiment

All animal experiments were approved by the Institutional Animal Care and Use Committee of Xi’an Medical University. The C57BL/6J mice were housed by 4 mice per cage with a 12 h light/dark cycle, 22 ± 1 °C and 55% humidity, with free access to food and water. The C57BL/6J mice were divided into the pyruvate group and the control group (10 mice per group). The blood samples (100 μL/mouse) were first collected from the mice via the tail vein, and then the mice in the pyruvate group were administered pyruvate via intraperitoneal injection (0.5 g/kg body weight in 0.1 mL PBS per time) 4 times with intervals of 2 h. The control mice were injected in the same way with PBS. Then, the mice were sacrificed for the collection of serum and liver. Liver tissues were used for total RNA extraction and protein extraction. Serums were used for the measurement of FGF21 and pyruvate levels.

### 4.10. Pyruvate Measurement

Pyruvate levels were measured using a colorimetric assay kit. Briefly, the plasma samples (50 μL per well) were incubated for 30 min at RT with the reaction mix (50 μL per well), which contained 40 μL pyruvate assay buffer, 2 μL pyruvate probe and 2 μL enzymes. Then, the OD values at 570 nm were measured to calculate pyruvate levels.

### 4.11. ALT and AST Activity Assay

ALT and AST activities in mouse serum were measured using the colorimetric assay kits according to the instruction. Briefly, the serum samples were first mixed with buffer solution and substrate buffer for 30 min at 37 °C. Chromogenic agent was then added for 20 min incubation at 37 °C. Finally, stop solution was added and the OD values at 510 nm were read for the calculation of AST and ALT activities according to the standard curve.

### 4.12. H&E Staining

The liver tissues were fixed by 4% paraformaldehyde and then routinely embedded by paraffin for the preparation of slides. H&E staining was carried out in slides according to the instruction, and the images were photographed.

### 4.13. Statistical Analysis

Data are expressed as means ± S.E.M. The D’Agostino–Pearson omnibus test was applied to test the data distribution normality. Comparisons of the means of multiple groups were analyzed via one-way ANOVA followed by Bonferroni post hoc tests for normally distributed data. *p* < 0.05 was considered to show a statistically significant difference.

## 5. Conclusions

Pyruvate activates PDEs to reduce intracellular cAMP and then inhibits the cAMP–Epac–CREB signaling pathway to upregulate FGF21 expression in hepatocytes.

## Data Availability

The data presented in this study are available on request from the corresponding author.
